# Impact of COVID-19 lockdown on food habits, appetite and body weight in Tunisian adults

**DOI:** 10.1017/jns.2022.58

**Published:** 2022-07-13

**Authors:** Saoussen Turki, Khaoula Bouzekri, Tarek Trabelsi, Jalila El Ati

**Affiliations:** 1 SURVEN (Nutrition Surveillance and Epidemiology in Tunisia) Research Laboratory, 11 Rue Jebel Lakhdar, bab Saadoun, 1007 Tunis, Tunisia; 2 High Institute of Medical Technologies, University Tunis El Manar, 9, Rue Docteur Zouheïr Safi, 1006 Tunis, Tunisia; 3 INNTA (National Institute of Nutrition and Food Technology), 11 Rue Jebel Lakhdar, bab Saadoun, 1007 Tunis, Tunisia

**Keywords:** Appetite, Body weight, COVID-19, Eating habits, Tunisian adults

## Abstract

Tunisia recorded the highest rate of COVID-19 positive cases and deaths in Africa but no studies assessed the impact of the pandemic on eating patterns as in the case of several countries. The objective of the present study was to investigate the perception of changes in food habits, appetite and body weight in Tunisian adults of both genders aged 20–74 years old. A cross-sectional study has been carried out with a non-probabilistic sampling method based on an online self-administered survey. Of overall 1082 adults included in the study, 57⋅8 % reported a change in their eating habits: 21⋅2 % an increase in their consumption of fresh fruits, vegetables, pulses, pasta and bread, while 36⋅6 % an increase of homemade cakes and biscuits, sweets, processed meat, sugary drinks and alcoholic drinks. In addition, tea, coffee and herbal tea have been reported as excessively consumed during the lockdown period. More than half experienced appetite variations (34⋅6 % increased appetite and 23⋅0 % appetite loss). Inequality detrimental to women was reported regarding eating habit changes (women consumed more unhealthier foods than men). Elderly subjects (over 60 years) were less likely to negatively change food habits in comparison with young adults (20–25 years), while ungraduated respondents were more prone to negatively change their food habits. Almost half reported weight gain. As the negative influence of the lockdown period on eating habits with the increase of obesity risk has been detected, health policy may be advised to focus on using mass media campaigns to promote healthy eating habits, in particular for illiterate and young people.

## Introduction

The COVID-19 pandemic has been considered as the most serious public health concern of the century since the second world war^(1)^. In fact, a new class of coronavirus, known as SARS-CoV-2 (severe acute respiratory syndrome coronavirus 2) has been discovered in December 2019 in China causing a new respiratory disease named COVID-19^(2)^.

The worldwide spread of the disease among human populations led to a global health crisis causing more than 6 358 899 deaths^(3)^. In Tunisia, the COVID-19 emerged first in March 2020. Overall, the country experienced five pandemic waves. On 29 July 2021, the country recorded the highest rate of positive cases and deaths in Africa with nearby 300 daily deaths^(4)^.

Prior to vaccines which is being now the best way to control the pandemic, lockdown measures have been adopted as a first virus spread preventive strategy. This strategy prevented public access to supermarkets, shops and recreation facilities. Hence, lockdown restricted outdoor physical activity as well as people's access to fresh food causing major dietary changes. Therefore, there had been great interest in literature on the impact of lockdown on dietary changes in various population groups. Over the two last years, many surveys were conducted online with the aim of studying eating habits and lifestyle changes during general confinement^(5–10)^. In a recent review, it has been shown that throughout Europe and worldwide, COVID-19 lockdown impacted dietary practices both negatively and positively^(11)^. In fact, results that have been reported among different countries were controversial with regards to consumption of alcohol, fresh produce such as fruits and vegetables as well as comfort foods including sweets, fried food, snack foods and processed foods^(11)^. In addition, we still do not know the consequence of the COVID-19 infection in terms of long-term symptoms. Due to the fact that smell/taste symptoms have been also involved in changes of food habits^(12–14)^.

Unlike European populations, scarce is known about the impact of confinement measures on the dietary habits in Tunisia. Research studies have focused mainly on issues related to changes in food purchasing behaviour and food preparation practices^(15)^ or food budget and waste^(16)^. Only one study included Tunisia among seventeen other countries from the Middle East and North Africa region to assess eating habits and lifestyle during the coronavirus 2019^(17)^. However, no conclusion can be ruled with this study since Tunisian nationals (*n* = 77) represented only 2⋅6 % of the studied population^(17)^. This work provides a first description of how the pandemic caused by COVID-19 changed the eating habits in Tunisia. This could help decision makers to better manage the public health consequences of the health crisis.

## Methods

### Participant's selection and study design

The study was carried out by SURVEN research team (Nutrition Surveillance and Epidemiology in Tunisia) from the National Institute of Nutrition and Food Technology in Tunisia. It is a cross-sectional study based on a self-administered questionnaire with a non-probabilistic sampling method that was conducted in adults of both genders aged 20–74 years old. In fact, based on studies^(5,6)^ conducted in Mediterranean countries, a Google Forms questionnaire was designed and disseminated to Tunisian adults through social networks (Facebook and Instagram) as well as institutional and private mailing lists. To calculate the sample size, the data published by the INS in 2021 were taken as reference. Tunisian population was estimated at 12,069,872 million inhabitants^(18)^. Accordingly, the calculation of the sample size was carried out with a 95 % confidence level and a 3 % of precision, since the expected proportion of the change in population was unknown, 0⋅5 proportion was selected. The theoretical sample size was 1067 subjects^(19)^. Participation in the study was completely free, voluntary and anonymous with the informed consent of the participants on the data sharing and confidentiality policy. No personal data has been requested, in accordance with the laws on the protection of personal data and the guarantee of digital rights. Therefore, this online survey does not require ethics committee approval.

### Questionnaire design

The questionnaire was established using Google Forms and sent in French and Arabic languages to meet the acceptance and understanding of the Tunisian population. The questionnaire was composed of three sections. *The first section* was dedicated to socio-demographic data collection. *The second section* included anthropometric and medical data collection. Weight and height were reported by the participants and used for the calculation of the body mass index (BMI) expressed as kg/m^2^. For BMI below 18⋅5, the participant was considered underweighted. For BMI between 18⋅5 and 24⋅9, the subject was classified as normal weighted. For BMI between 25 and 29⋅9, the subject was considered as pre-obese, and if BMI exceeded 30, the subject was considered as obese. Data describing the general health status have been recorded. Subjects suffering from multiple chronic diseases (more than three disease types) were considered at morbid health status. *The third section* included a retrospective report of changes in eating habits during the period of lockdown. We used the results of previous survey conducted among Tunisian adults to assess their food consumption, in particular the list of food items the most consumed and considered as healthy or unhealthy^(20)^. This section was useful to calculate scores of eating habits improvement (EHI score) and deterioration (EHD score). In fact, for an increase in consumption of healthy food items (fresh fruits, vegetables, pulses, nuts, pasta and bread, fish, poultry, meat) and/or a decrease in unhealthy food intake (industrial cakes and biscuits, homemade pastries, chocolate and sweets, processed meat, snacks, sugary drinks, alcoholic drinks) (positive change), one point was attributed to EHI score. Reversely, one point was attributed to EHD score if participants said increasing their consumption of unhealthy foods and/or decreasing healthy food intake (negative change). The consumption of natural stimulants such as tea, coffee and herbal tea was also assessed. Finally, participants were asked for sport practice frequency during the lockdown period. A version of the online questionnaire (translated in English) is available in Supplementary Material S1.

Prior to survey launch, a pilot study has been carried out on forty subjects to assess the clarity and the acceptance of the questionnaire. Feedback has been collected to improve questionnaire quality-content.

### Data collection and statistical analysis

The participants filled in the forms directly connected to the Google platform. Once completed, each response was sent to this platform and the final database was uploaded as a Microsoft Excel sheet. The questionnaire was disseminated over 2 months (between 17 May and 20 July 2021); this period coincided with the start of the fourth wave of COVID-19 in Tunisia. No general lockdown measure has been instigated during that period, but intercity movements were prohibited, and a curfew was imposed from 10 pm to 5 am.

Descriptive results are expressed as means for continuous variables, and as proportions for categorical variables. Shapiro–Wilk and Skewness–Kurtosis tests were performed to assess normality. The *χ*^2^ and Fisher's exact tests were used to assess distribution equality between groups. In contrast, Mann–Whitney and Kruskal–Wallis *U* tests were applied to compare continuous variables between two or more groups, respectively, when normality was not confirmed. Where relevant, *post hoc* tests were applied for more precise analyses. The Spearman correlation coefficient was calculated for the associated continuous variables. The association between independent variable coded as two category response variables (virus infection or exposure) and socio-demographic variables (age, sex, region, education, professional activity) was assessed using binary logistic regression models (odds ratio, OR). For independent variable coded as more than two category response variables (perceived changes in eating habits, appetite, weight), we used multinomial logistic regression models (relative risk ratio, RRR) to estimate the association with socio-demographic co-variates. The type I error risk was set at 0⋅05 for all analyses. Statistical analyses were performed using Stata (StataCorp. Stata Statistical Software: Release 14.0. College Station, TX: StataCorp LP. 2015)^(21)^.

## Results

### Characteristics of the participants

According to the sample size calculation, 1067 people are expected to respond to our survey. As the survey was conducted online, 1121 participants filled the questionnaire, 39 questionnaires were rejected because they were from ineligible people (age under 20 years) and 1089 were validated. Participants were classified into three groups according to their age: young adult (age 20–25 years), adults (26–60 years) and elderly (over 60 years).

### Socio-demographic data

As shown in Table 1, women represented 74⋅3 % of the population. Adults were the most predominant group for both genders. Elderly subjects were the least represented group in the sample. The study sample covered all regions of Tunisia, with comparable distribution for both genders.

**Table 1.** Socio-demographic characteristics of the participants

	Total population *n* (%)^a^	Women *n* (%)	Men *n* (%)
	1082 (100)	804 (74⋅3)	278 (25⋅7)
Habitation region		P = 0·192*	
Greater Tunis	482 (44⋅6)	350 (43⋅5)	132 (47⋅5)
North-East	154 (14⋅2)	120 (14⋅9)	34 (12⋅2)
North-West	93 (8⋅6)	72 (9⋅0)	21 (7⋅5)
Centre-East	195 (18⋅0)	151 (18⋅8)	44 (15⋅8)
Centre-West	44 (4⋅1)	31 (3⋅9)	13 (4⋅7)
South-East	90 (8⋅3)	67 (8⋅3)	23 (8⋅3)
South-West	24 (2⋅2)	13 (1⋅6)	11 (4⋅0)
Age category		P < 0·0001*	
Young adult (20–25 years)	339 (31⋅3)	277 (34⋅4)	62 (22⋅3)
Adults (25–60 years)	691 (63⋅9)	503 (62⋅6)	188 (67⋅6)
Elderly (60 years and over)	52 (4⋅8)	24 (2⋅9)	28 (10⋅1)
Graduation status		P < 0·0001*	
Non-graduated	4 (0⋅4)	1 (0⋅1)	3 (1⋅1)
Primary school graduation	9 (0⋅8)	6 (0⋅7)	3 (1⋅1)
Secondary school graduation	76 (7⋅0)	37 (4⋅6)	39 (14⋅0)
University graduation	993 (91⋅8)	760 (94⋅5)	233 (83⋅8)
Occupational status		P < 0·0001*	
Unemployed or Housewives	145 (13⋅4)	131 (16⋅3)	14 (0⋅5)
Student	423 (39⋅1)	346 (43⋅0)	77 (27⋅7)
Worker	71 (6⋅6)	40 (5⋅0)	31 (11⋅1)
Intermediate executive	132 (12⋅2)	84 (10⋅4)	48 (17⋅3)
Upper executive	272 (25⋅1)	186 (23⋅1)	86 (30⋅9)
Retiree	39 (3⋅6)	17 (2⋅1)	39 (7⋅9)
Household size (*N* = 1059)^b^		P = 0·108*	
Living alone	29 (2⋅7)	17 (2⋅1)	12 (4⋅3)
Small size family (≤4 members)	798 (73⋅8)	604 (75⋅1)	194 (69⋅8)
Large size family (>4 members)	232 (21⋅4)	171 (21⋅3)	61 (21⋅9)
Unspecified	23 (2⋅1)	12 (1⋅5)	11 (4⋅0)

a Data are expressed as the total number of respondents with percentages between brackets (%).

b Number of respondents specifying their household size.

* *P*-value: null hypothesis of same distribution between both genders (*χ*^2^ and Fisher exact test); null hypothesis rejected at *P* < 0⋅05.

### Anthropometric and medical data

Data regarding anthropometric measurements and the participant health status are summarised in Table 2. Mean age of the sample was 32⋅5 ± 12 years. BMI was calculated, 48⋅8 % of respondents had a normal weight, 31⋅1 % were pre-obese and 14⋅5 % were obese, with no gender difference (Table 2). Most of the respondents (78⋅7 %) did not declare any chronic illness. For the rest, the most cited diseases were endocrine, metabolic, haematological and cardiovascular diseases. The great majority of the respondents reported spontaneous eating with no special diet. For COVID-19 incidence, nearby 68 % of the respondents confirmed not being infected with SARS-CoV-2 at the moment of the survey, 15⋅4 % confirmed to have contracted the disease in its mild form, 1⋅2 % experienced a severe form of COVID-19 with no gender difference (*P* = 0⋅558). Only 21⋅7 % declared being vaccinated against the virus during the survey period (Table 2).

**Table 2.** Anthropometrics and medical data of the participants

	Total population *n* (%)^a^		Women *n* (%)		Men *n* (%)	
	1082 (100)		804 (74·3)		278 (25·7)	
Age (years)^b^	32·5	12·0	31·1	10·8	36·5	14·2
Weight (kg)^b^	71·1	15·2	67·8	13·9	80·5	14·8
Height (m)^b^	1·68	0·08	1·64	0·06	1·77	0·06
BMI (kg/m^2^)^b^	25·1	4·9	25	5·0	25·4	4·4
Nutritional status^a^	P = 0·2*					
Underweight	60 (5·5)		50 (6·2)		10 (3·6)	
Normal weight	528 (48·8)		396 (49·2)		132 (47·5)	
Pre-obesity	337 (31·1)		241 (30·0)		96 (34·5)	
Obesity	157 (14·5)		117 (14·6)		40 (14·4)	
History of Chronic diseases (number of diseases)^a^	P = 0·2*					
No chronic disease	852 (78·7)		632 (78·6)		220 (79·1)	
One disease	195 (18·0)		143 (17·8)		52 (18·7)	
Multiple diseases	35 (3·2)		29 (3·6)		6 (2·1)	
History of chronic diseases (type of diseases)^a^	P = 0·002*					
Cardiovascular diseases	25 (10·9)		14 (8·1)		11 (19·0)	
Endocrine, nutritional or metabolic diseases	56 (24·3)		37 (21·5)		19 (32·7)	
Respiratory and ENT diseases	14 (6·1)		8 (4·6)		6 (10·3)	
Digestive diseases	13 (5·7)		12 (7·0)		1 (1·7)	
Haematological diseases	26 (11·3)		26 (15·1)		0 (0·0)	
Genecology, urinary and kidney diseases	9 (3·9)		5 (2·9)		4 (6·8)	
Cancer	2 (0·9)		2 (1·2)		0 (0·0)	
Other diseases	50 (21·7)		39 (22·7)		11 (19·0)	
Morbid health status (more than three diseases)	35 (15·2)		29 (16·9)		6 (10·3)	
Diet^a^	P = 0·004*					
Spontaneous feeding with no diet	960 (88·7)		713 (88·7)		247 (88·8)	
Healthy diet	23 (2·1)		15 (3·2)		8 (1·4)	
Weight-loss diet	34 (3·1)		29 (3·6)		5 (1·8)	
Reduced sugar/salt diet	20 (1·8)		8 (1·0)		12 (4·3)	
Therapeutic diet	13 (1·2)		11 (1·4)		2 (0·7)	
Vegetarian diet	2 (0·2)		2 (0·2)		0 (0·0)	
Not specified	30 (2·8)		26 (3·2)		4 (1·4)	
Incidence of COVID-19^a^	P = 0·55*					
Not exposed	734 (67·8)		539 (67·0)		195 (70·1)	
Suspected infection	168 (15·5)		132 (16·4)		36 (12·9)	
Mid-form infection	167 (15·4)		124 (15·4)		43 (15·5)	
Severe form infection	13 (1·2)		9 (1·1)		4 (1·4)	
Occurrence of family death due to COVID-19^a^	P = 0·90*					
No	886 (81·9)		659 (82·0)		227 (81·6)	
Yes	196 (18·1)		145 (18·0)		51 (18·4)	
Vaccination against COVID-19^a^	P = 0·14*					
No	847 (78·3)		638 (79·3)		209 (75·2)	
Yes	235 (21·7)		166 (20·7)		69 (24·8)	

a Data are expressed as the total number of respondents with proportions between brackets for categorical variables.

b Data are expressed as mean with standard deviation in separate columns.

* *P*-value: null hypothesis of same distribution between both genders (*χ*^2^ and Fisher exact test); null hypothesis rejected at *P* < 0·05.

### Perception of eating habits changes during lockdown periods

As shown in Table 3, during lockdown periods, 42⋅1 % of the respondents declared no change in eating habits. However, 21⋅2 % reported an increase of their consumption of fresh fruits, vegetables, pulses, pasta and bread (positive change), while 36⋅6 % an increase of homemade cakes and biscuits, sweets, processed meat, sugary drinks and alcoholic drinks (negative change) (Table 3). In parallel, nearby 45 % reported no change in the number of meals consumed per day, 16 % declared skipping a main meal, while only 5⋅4 % reported skipping snacks. Meals and snack's addition had been reported by 7⋅7 and 9⋅2 % of the participants, respectively. Excess snacking was declared by 16⋅3 %.

**Table 3.** Perception of eating habits, appetite and body weight changes during COVID-19 lockdown periods

	Total population *n* (%)^a^	Women *n* (%)	Men *n* (%)
	1082 (100)	804 (74⋅3)	278 (25⋅7)
Eating habits change^a^		*P* < 0⋅001*	
No change	456 (42⋅1)	315 (39⋅2)	141 (50⋅7)
Negative change^b^	396 (36⋅6)	322 (40)	74 (26⋅6)
Positive change^c^	230 (21⋅2)	167 (20⋅8)	63 (22⋅6)
Appetite change^a^		*P* < 0⋅001*	
Stable appetite	459 (42⋅4)	306 (38⋅1)	153 (55⋅0)
Appetite loss	249 (23⋅0)	182 (22⋅6)	67 (24⋅1)
Appetite gain	374 (34⋅6)	316 (39⋅3)	58 (20⋅9)
Weight change^a^		*P* < 0⋅001*	
Stable weight	385 (35⋅6)	258 (32⋅1)	127 (45⋅7)
Weight loss	176 (16⋅3)	129 (16⋅0)	47 (16⋅9)
Slight weight gain	374 (34⋅6)	289 (35⋅9)	85 (30⋅6)
Noticeable weight gain	147 (13⋅6)	128 (15⋅9)	19 (6⋅8)
Change in number of meals^a^		*P* < 0⋅001*	
No change	491 (45⋅4)	333 (41⋅4)	158 (56⋅8)
Skipping main meal	174 (16⋅0)	136 (16⋅9)	38 (13⋅7)
Skipping snacks	58 (5⋅4)	44 (5⋅5)	14 (5⋅0)
Main meal addition	83 (7⋅7)	64 (8⋅0)	19 (6⋅8)
Snacks addition	100 (9⋅2)	81 (10⋅1)	19 (6⋅8)
Excessive snacking	176 (16⋅3)	146 (18⋅2)	30 (10⋅8)
Consumption of supplementary foods^a^		*P* < 0⋅001*	
No	756 (69⋅9)	527 (65⋅5)	229 (82⋅4)
Yes	316 (29⋅2)	270 (33⋅6)	46 (16⋅5)
Not specified	10 (0⋅9)	7 (0⋅9)	3 (1⋅1)
Nutrition information search^a^		*P* = 0⋅0001*	
No search	500 (46⋅2)	343 (42⋅7)	157 (56⋅5)
Search in media (TV, Radio, … )	214 (19⋅8)	166 (20⋅7)	48 (17⋅3)
Search in social media	294 (27⋅2)	232 (28⋅9)	62 (22⋅3)
Consulting nutrition expert	65 (6⋅0)	57 (7⋅1)	8 (2⋅9)
Not specified	9 (0⋅8)	5 (0⋅6)	4 (1⋅4)
Sport practice frequency		*P* < 0⋅001*	
No sport practice	675 (62⋅5)	529 (65⋅7)	146 (52⋅5)
1 to 2 times per week	235 (21⋅7)	164 (20⋅4)	71 (25⋅5)
3 to 4 times per week	125 (11⋅5)	84 (10⋅5)	41 (14⋅8)
More than 5 times per week	47 (4⋅3)	27 (3⋅4)	20 (7⋅2)

a Data are expressed as the total number of respondents with percentages between brackets (%).

b Negative change refers to an increase in consumption of homemade cakes and biscuits, sweets, processed meat, sugary and alcoholic drinks.

c Positive change refers to an increase in consumption of fresh fruits, fish, vegetables, pulses, pasta and bread.

* *P*-value: null hypothesis of same distribution between both genders (*χ*^2^ and Fisher exact test); null hypothesis rejected at *P* < 0⋅05.

With the onset of the pandemic, 29⋅2 % consumed supplementary foods. According to our study, virus exposure or infection would have increased the consumption of dietary supplements (virus exposure: OR 2⋅28; confidence interval (95 % CI) 1⋅58, 3⋅30; *P* < 0⋅001, virus infection: OR 3⋅60; 95 % CI 2⋅53, 5⋅12; *P* < 0⋅001).

Besides, gender significant differences (*P* < 0⋅001) were noted for eating habit changes as well as the number of meals per day variation (Table 3). Our results showed that the COVID-19 pandemic impacted the eating habits of women negatively as compared to men. Indeed, the percentage of women (40 %) varying negatively their eating habits was significantly higher than men (26⋅6 %). On the other side, 50⋅7 % of men confirmed no change in eating habits *v*. 39⋅2 % of women. The logistic regression test showed that men were less likely to negatively vary their eating habits relative to women (RRR 0⋅5; 95 % CI 0⋅36, 0⋅7; *P* < 0⋅001).

For changes in the number of daily meals during periods of confinement, women reported a greater tendency to over snack (18⋅2 %) than men (10⋅8 %). Logistic regression analysis showed that compared to women, men were less likely to skip meals (RRR  0⋅60; 95 % CI 0⋅39, 0⋅91; *P* = 0⋅018), to add snacks (RRR 0⋅52; 95 % CI 0⋅3, 0⋅9; *P* = 0⋅020) and to snack excessively (RRR 0⋅42; 95 % CI 0⋅27, 0⋅66; *P* < 0⋅001).

Furthermore, Kruskal–Wallis test results showed that age factor made a significant difference in perception of eating habits change (*P* = 0⋅012). In fact, as shown in Table 3, for 63⋅5 % of the elderly, eating habits were stable compared to young adults (41 %). These latter ones reported more tendency to negatively change their eating habits (43⋅4 %) compared to adults (34⋅6 %) and elderly (19⋅2 %). Adults were more prevalent (24⋅3 %) in reporting a positive change in eating habits as compared to young adults (15⋅6 %) and elderly (17⋅3 %). The multinomial regression test showed that the elderly subjects were less likely to negatively change food habits in comparison with young adults (RRR 0⋅27; 95 % CI 0⋅12, 0⋅58; *P* = 0⋅001), while adults were more likely to vary their habits positively (RRR 1⋅49; 95 % CI 1⋅02, 2⋅16; *P* = 0⋅035) than the younger.

Graduation was also found to make a significant difference (*P* = 0⋅0106) for the eating habits change. In fact, 75 % of ungraduated respondents were more prone to negatively change their eating habits against 44⋅4 and 31⋅6 % for primary and secondary-graduated participants. Reversely, graduated respondents reported positive change in eating habits as the following: 33⋅3 % for primary level, 10⋅5 % for secondary level, 22⋅0 % for university level, whereas none of the ungraduated participants reported a positive change of eating habits.

Finally, our results showed that household size made a significant difference (*P* = 0⋅039) in eating habit changes. Among those who specified their household's size (1059 participants), subjects declaring no change in eating habits during the periods of confinement were more prevalent among large households (number of people >4) with a percentage of 48⋅7 %. This percentage is higher than the percentage of those living in small-sized households (40⋅7 %) as well as those living alone (37⋅9 %). The latter ones were mostly perceived to vary their eating habits negatively (Table 3). On the other hand, participants belonging to small-sized households were prevalent to shift positively their eating habits during confinement (22⋅4 %) in comparison with solitary people (17⋅2 %) and large households (16⋅3 %).

With regards to health parameters, skipping meals during confinement periods was observed at higher percentage among the underweight class (23 %) and at lower percentage for pre-obese subjects (12⋅2 %). Excessive snacking was more reported by obese (22⋅3 %), and pre-obese (19⋅6 %) subjects compared to normal (13 %) and underweighted (10 %) subjects. Moreover, the population reporting stability in number of daily meals were predominantly among those following a healthy diet (52⋅1 %) and a diet reduced in salt and sugar (55 %). Surprisingly, those who reported being on a weight loss or therapeutic diet were most prone to add daily meals and snacks.

### Perception of appetite changes during lockdown periods

With regards to appetite change, 42⋅4 % of our participants declared no appetite change, 23⋅0 % perceived a loss of appetite and 34⋅6 % a gain of appetite (Table 3). Gender significant differences (*P* < 0⋅001) were noted as well for perception of appetite change. Indeed, a higher percentage of women (39⋅9 %) declared gaining appetite as compared to men (20⋅9 %). In addition, 55⋅0 % of men reported no change in appetite *v*. 38⋅1 % of women.

Multinomial logistic regression showed that men were less likely to perceive gain appetite as compared to women (RRR 0⋅42, 95 % CI 0⋅29, 0⋅6; *P* < 0⋅001).

Kruskal–Wallis test results showed that age factor also made a significant difference for perception of appetite variation (*P* < 0⋅001). In fact, those who reported no change in appetite were mostly elderly (82⋅7 %) compared to young adults (34⋅1 %) and adults (43⋅3 %). On the other hand, those who declared loss of appetite were predominantly young adults and adults (24⋅8 and 22⋅9 %, respectively) *v*. 13⋅5 % for the elderly. Finally, young adults reported a gain appetite with a higher percentage (40⋅7 %) than adults (33⋅9 %) and elderly (3⋅8 %). The multinomial logistic regression test proved that elderly respondents were less willing to lose their appetite during periods of confinement compared to young adults (RRR 0⋅13; 95 % CI 0⋅049, 0⋅35; *P* < 0⋅001). Adults and elderly were also less willing to have an appetite gain relative to youth (adults: RRR 0⋅52; 95 % CI 0⋅38, 0⋅72; *P* < 0⋅001, elderly: RRR 0⋅011; 95 % CI 0⋅001, 0⋅086; *P* < 0⋅001).

Furthermore, our results showed that professional status made a significant difference (*P* < 0⋅001) in appetite change. In fact, no change in appetite was mostly reported by retired respondents (84⋅6 %) as compared to students (38⋅5 %) and workers (46⋅5 %). Gain appetite was predominantly perceived among unemployed and housewives’ subgroups as well as students with percentages of 39⋅3 and 38⋅8 %, respectively. Logistic regression showed that the retired respondents were less likely to change appetite either by loss (RRR 0⋅22, 95 % CI 0⋅06, 0⋅8; *P* = 0⋅024) or by gain (RRR 0⋅13; 95 % CI 0⋅038, 0⋅047; *P* = 0⋅002) in comparison with unemployed and housewives’ subgroup.

Moreover, underweighted subjects declared mostly no change in appetite (55⋅0 %) as compared to other BMI classes (44⋅7 % for subjects with normal weight, 38⋅9 % for obese and 38, 3 % for pre-obese). Conversely, appetite gain was preponderant among obese (40⋅1 %) or pre-obese (40⋅5 %) compared to subjects with normal weight (30⋅7 %) and underweighted subjects (18⋅3 %). Logistic regression test showed that normal weighted, pre-obese and obese subjects were more willing to gain appetite during confinement compared to underweighted (normal weight: RRR 2⋅21; 95 % CI 1⋅07, 4⋅6; *P* = 0⋅032, pre-obesity: RRR 3⋅76; 95 % CI 1⋅77, 7⋅97; *P* = 0⋅001, obesity: RRR 3⋅9; 95 % CI 1⋅74, 8⋅71; *P* = 0⋅001).

### Perceptions of body weight changes during lockdown periods

Regarding perception of body weight change, 35⋅6 % reported weight stability, 16⋅3 % reported losing body weight *v*. 48⋅2 % reporting body weight gain (Table 3). Similarly, to eating habits and appetite change, gender significant differences (*P* < 0⋅001) were found for perception of body weight change. In fact, a higher percentage of women participants reported a significant body weight gain (15⋅9 %) as compared to men (6⋅8 %). Moreover, body weight stability was reported by 45⋅7 % of men participants *v*. 32⋅1 % of women. The test of logistic regression showed that men have lower risk of notably gaining body weight (RRR 0⋅34; 95 % CI 0⋅19, 0⋅59; *P* < 0⋅001) comparatively to women. According to age factor, a significant difference (*P* < 0⋅001) in perception of body weight change was found. For elderly, 57⋅7 % of the respondents perceived body weight stability during periods of confinement against 35⋅6 % for adults and 32⋅2 % for young adults. The body weight loss affected more significantly elderly subjects (23⋅1 %) compared to adults (15⋅6 %) and young adults (16⋅5 %). By contrast, young adults and adults had tendency to gain weight in higher percentage than elderly as shown in Table 3. The logistic regression test highlighted that compared to young adults, adults were less likely to gain weight slightly (RRR 0⋅64; 95 % CI 0⋅46, 0⋅89; *P* = 0⋅009) and significantly (RRR 0⋅55; 95 % CI 0⋅34, 0⋅88; *P* = 0⋅013). Similarly, elderly subjects were at lower risk of weight gain (RRR 0⋅15; 95 % CI 0⋅069, 0⋅36; *P* < 0⋅001). In agreement with those results, the Spearman test confirmed a negative significant association between age and perception of body weight change during confinement periods (*r* = −0⋅075, *P* = 0⋅013). Employment status made a difference statistically significant for perception of body weight change (*P* = 0⋅001). In fact, 61⋅5 % of the retired subgroup reported a weight stability against only 29⋅7 % for the unemployed and housewives’ subgroup and 40⋅0 % for upper executives. Those who reported noticeable weight gain were for the greater majority among the class of unemployed. The multinomial regression test showed a low risk of slight weight gain for the subgroup of retirees (RRR 0⋅33; 95 % CI 0⋅13, 0⋅85; *P* = 0⋅022) in comparison to unemployed and housewives’ subgroup. The retired subgroup remained as well less exposed to noticeable weight gain (RRR 0⋅092; 95 % CI 0⋅011, 0⋅74; *P* = 0⋅025) along with the subgroup of senior executives (RRR 0⋅45; 95 % CI 0⋅24, 0⋅86; *P* = 0⋅016) as compared to unemployed subjects.

Along with previously cited socio-demographic parameters, nutritional status made a difference statistically significant for the body weight change (*P* < 0⋅001). In fact, a stable weight was predominantly reported by underweighted (48⋅3 %) and normal weighted (43⋅8 %) subjects compared to pre-obese (27⋅3 %) and obese (21 %). However, noticeable weight gain was reported at higher percentage among obese subjects (29⋅9 %) as well as pre-obese (20⋅8 %) compared to subjects with a normal weight (5⋅7 %) and underweighted (0 %). The latter ones were rather the population most affected by body weight loss with a percentage of 36⋅7 % *v*. 17⋅2 % for normal weight, 13 % for pre-obese and 12⋅1 % for obese.

The logistic regression test showed that relative to underweighted, the risk of weight gain increased proportionally with BMI (normal BMI: RRR 2⋅56; 95 % CI 1⋅17, 5⋅60; *P* = 0⋅018, pre-obese: RRR 5⋅46; 95 % CI 2⋅42, 12⋅31; *P* < 0⋅001, obese: RRR 6⋅97; 95 % CI 2⋅87, 16⋅89; *P* < 0⋅001). Spearman's test confirmed the positive and statistically significant association between body weight change perception and BMI class (*r* = 0⋅30, *P* < 0⋅001). In addition, the health status had a significant impact on body weight change perception (*P* = 0⋅0213). Having a morbid health status did not favour weight stability, as the percentage of those reporting stable weight was low in subjects with morbid health status (11⋅4 %) compared to healthy subjects (35⋅7 %) or those with a single chronic disease (39⋅5 %). Morbid health status rather seems to favour weight change either by loss (28⋅6 % *v*. 14⋅9 % for healthy subjects) or by gain (57 % weight gain was reported for subjects with morbid health status *v*. 49⋅4 % for healthy subjects). The logistic regression test showed that having a morbid health status increased the risk of losing weight (RRR 6⋅6; 95 % CI 1⋅92, 22⋅62; *P* = 0⋅003) or gaining it significantly (RRR 6⋅77; 95 % CI 1⋅82, 25⋅20; *P* = 0⋅004) as compared to healthy participants. In addition, the association between weight gain and changes in eating habits was assessed. The logistic regression test confirmed that as compared to those who did not change their eating habits, increasing consumption of unhealthy food and decreasing consumption of healthy food (negative change) increased the risk of gaining weight slightly (RRR 5⋅62; 95 % CI 3⋅89, 8⋅09; *P* < 0⋅001) and notably (RRR 18⋅5; 95 % CI 10⋅93, 31⋅28; *P* < 0⋅001). Physical activity during lockdown period was also assessed. Most of the participants (62⋅5 %) were at sedentary status. Practicing physical activity more than five times a week during lockdown period presented an inverse association with body weight increase (RRR 0⋅22; 95 % CI 0⋅05, 0⋅97; *P* = 0⋅046).

### Perception of food consumption change during confinement periods

**Fig. 1.**
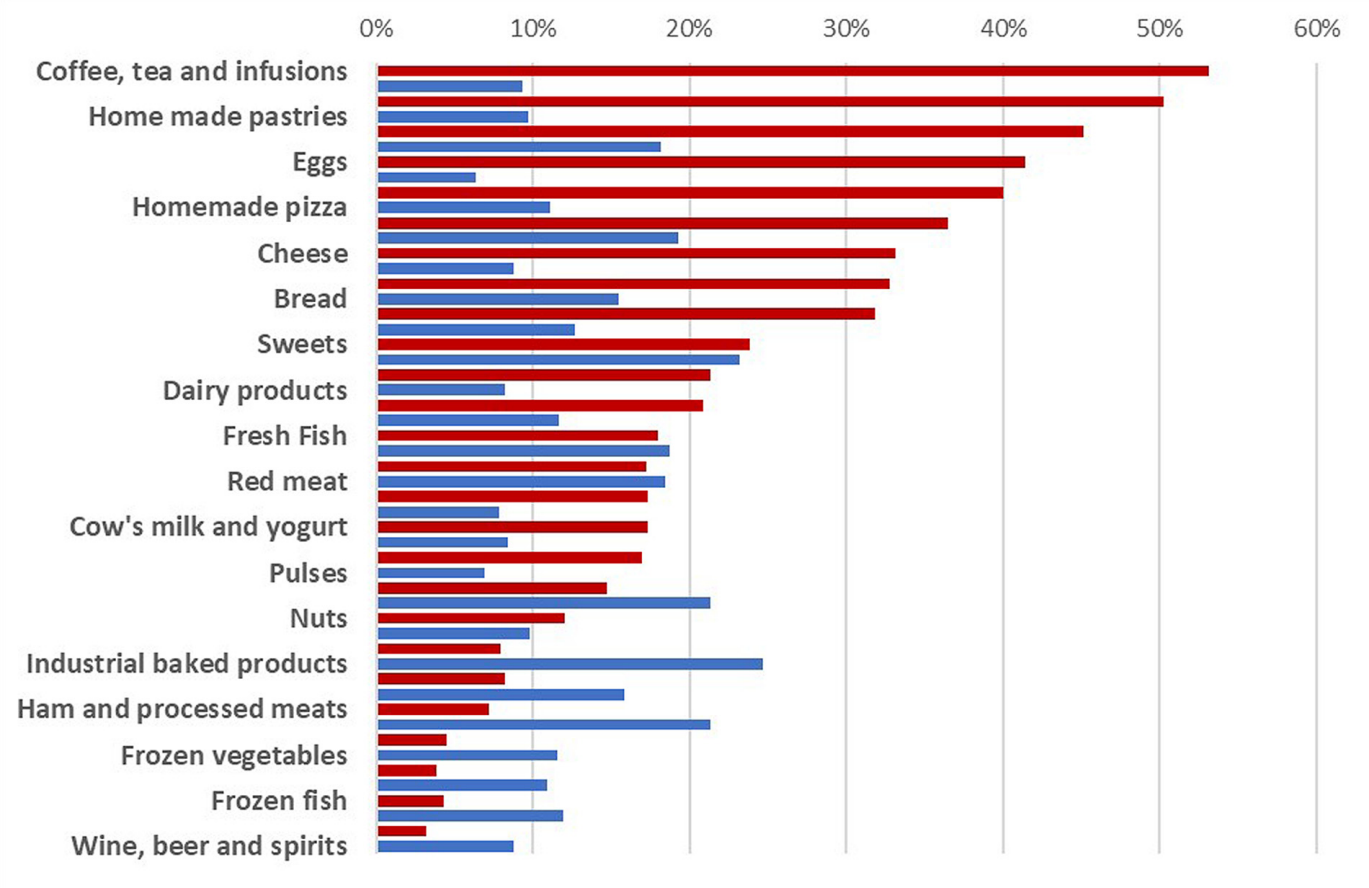
List of foods that consumption increased (red bars) and decreased (blue bars) during COVID-19 lockdown periods. Results are expressed as frequencies of food citation.

According to Fig. 1, foods that consumption had increased during the periods of confinement were coffee, tea and herbal tea, homemade pastries, fruit, eggs, fresh vegetables, cheese, bread, pasta and cereals. On the other hand, the foods that consumption was reduced were bakery products, sweets, ham and processed meat as well as sweet beverages.

**Fig. 2.**
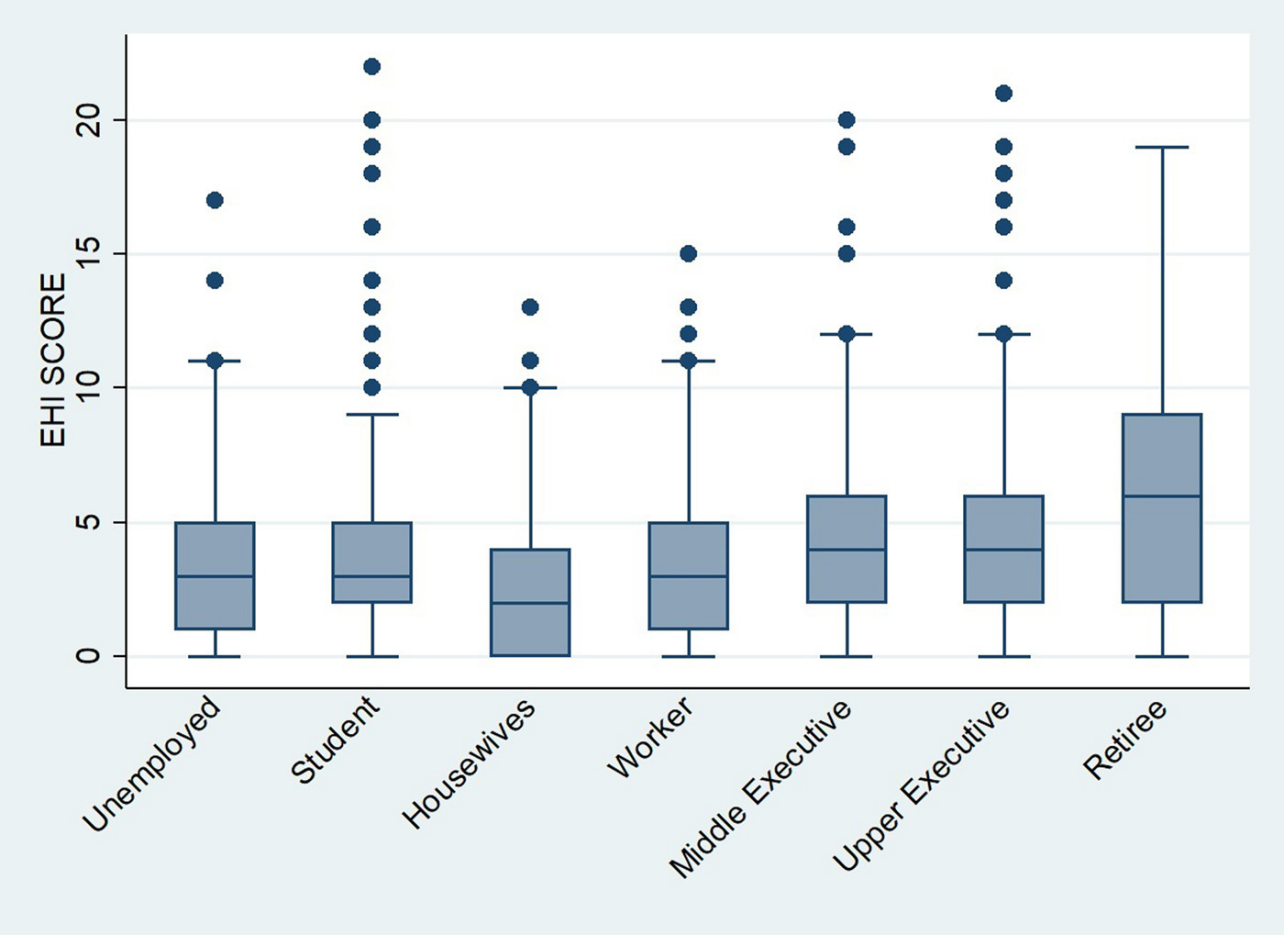
Eating habits improvement score (EHI score). Distribution over occupational status.

**Fig. 3.**
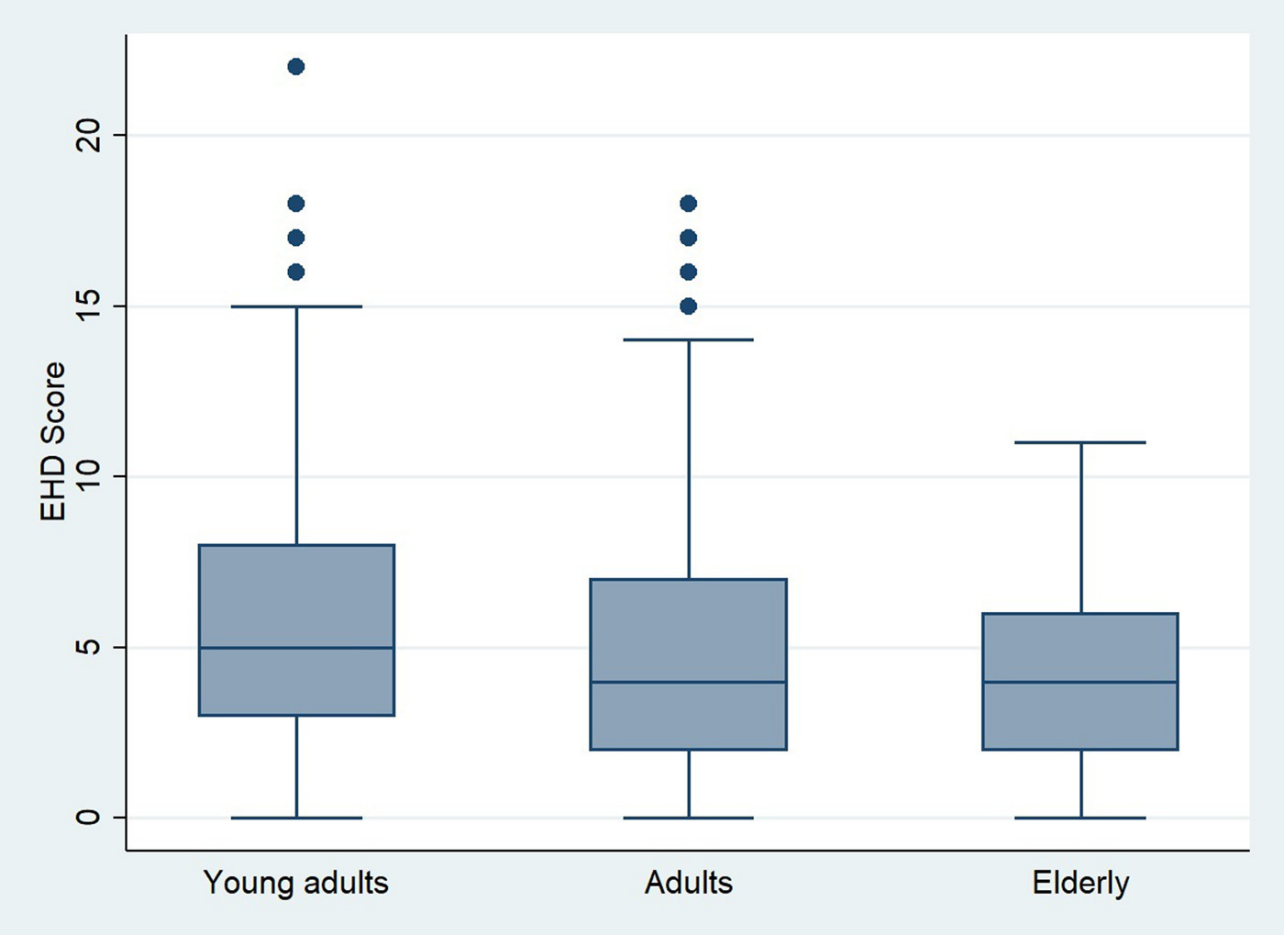
Eating habits deterioration score (EHD score). Distribution over age category.

**Fig. 4.**
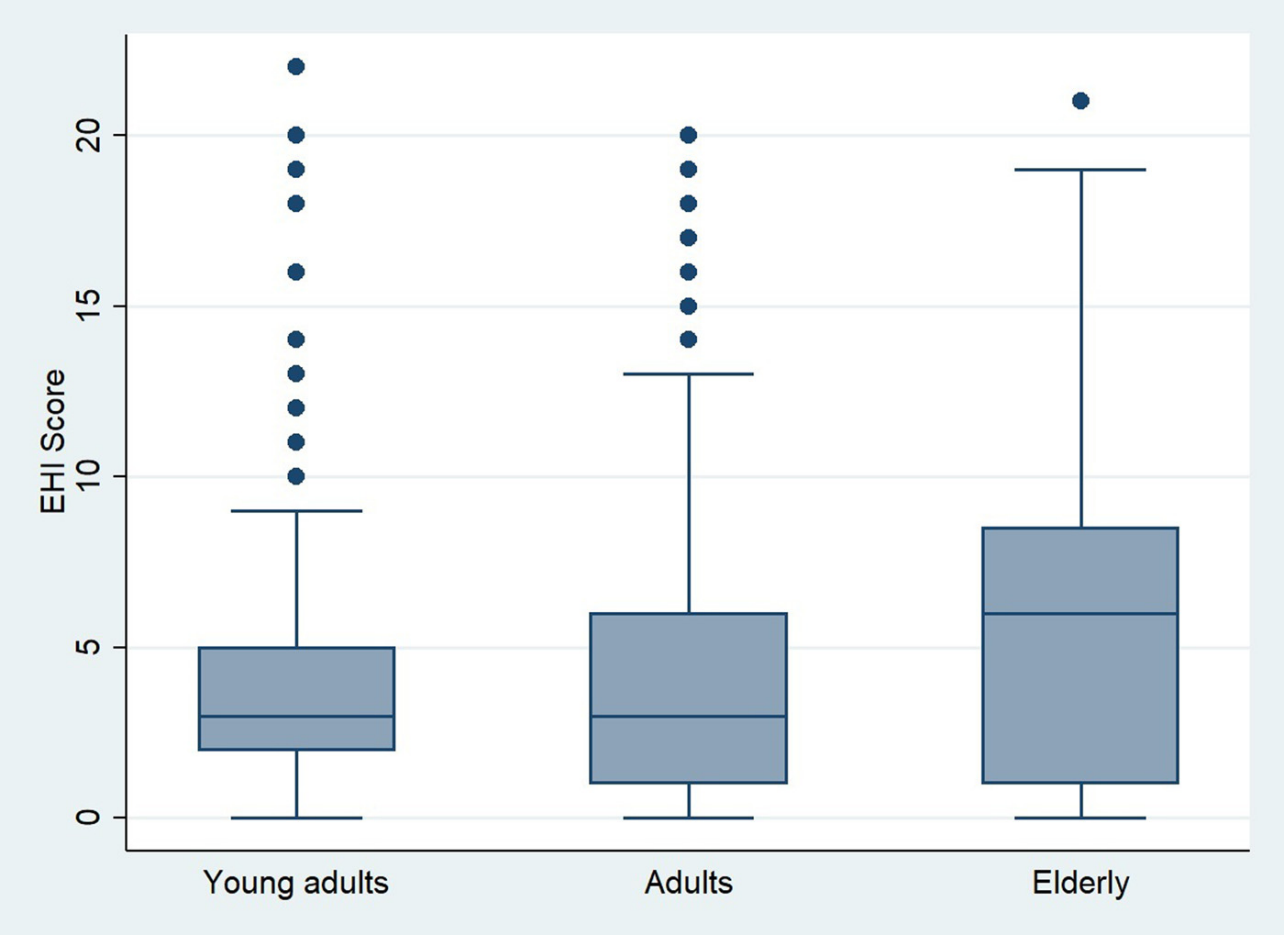
Eating habits improvement score (EHI score). Distribution over age category.

Furthermore, with regards to socio-demographic parameters, our results showed that both EHI and EHD scores were associated neither with gender, nor with habitation zone, graduation status or household size. However, professional status had a significant influence (*P* < 0⋅001) on the EHI. Indeed, as shown in Fig. 2, we noticed that the subgroup of retirees has the highest average of EHI while the lowest scores were prevalent among unemployed and housewives’ subgroup. But no significant difference (*P* = 0⋅06) was found for EHD score. For this latter score, Spearman test showed a negative significant association between age and EHD score (*r* = −0⋅106, *P* = 0⋅0005). According to Fig. 3, average EHD score was higher for young adults than adults and elderly. Reversely, as shown in Fig. 4, elderly scored higher EHI as compared to young, and adults’ respondents.

**Fig. 5.**
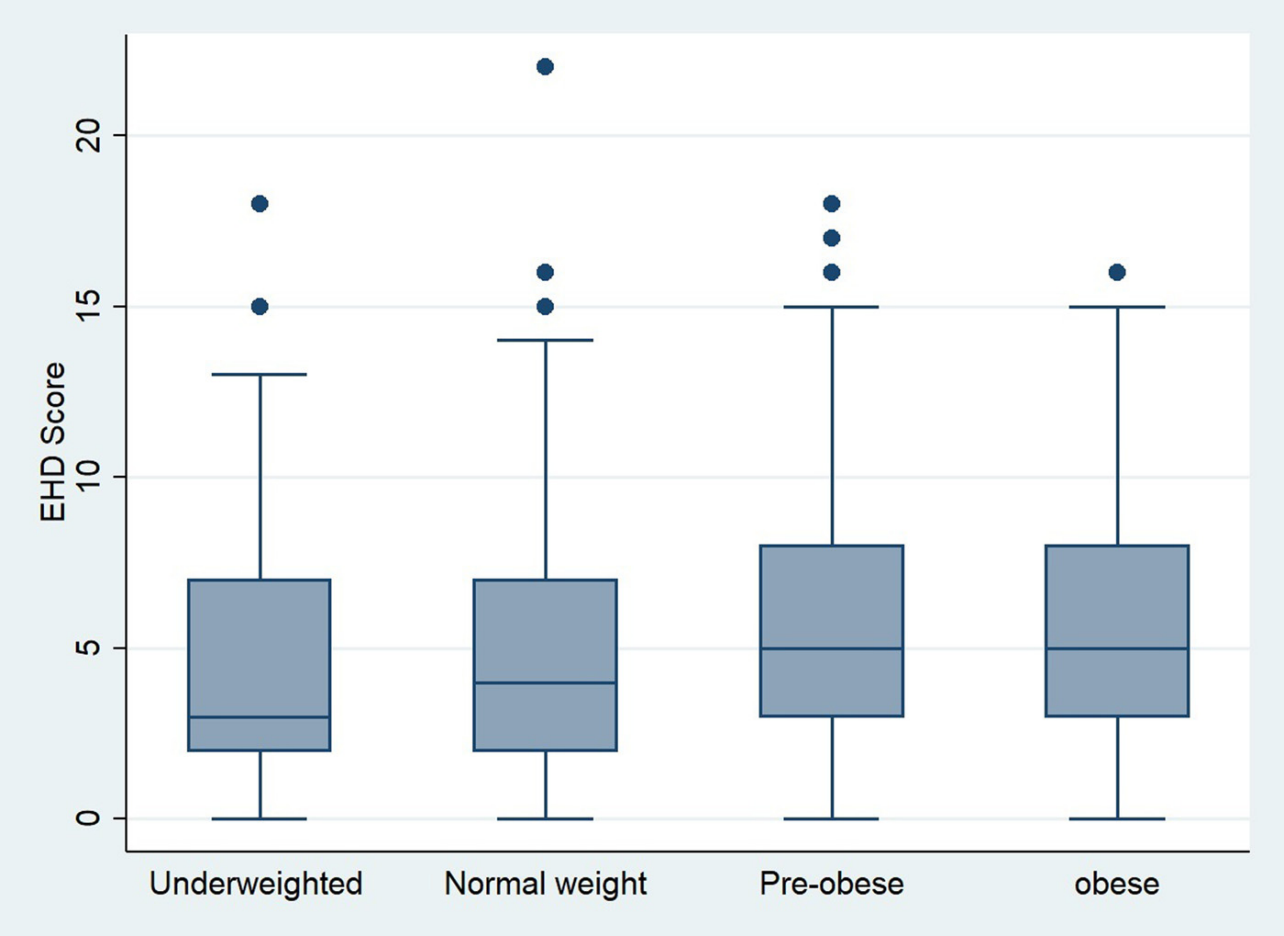
Eating habits deterioration score (EHD score). Distribution over BMI class.

According to anthropometric and medical parameters, Fig. 5 showed that the highest EHD were noticed for pre-obese and obese subjects. Finally, it should be stressed out that the Spearman test showed a positive significant association between EHD and increased appetite (*r* = 0⋅32, *P* < 0⋅001). Similarly, a significant positive association was found with body weight change (*r* = 0⋅27, *P* < 0⋅001). However, the test indicated a negative significant association between EHI and body weight change (*r* = −0⋅09, *P* = 0⋅0027).

## Discussion

The present study investigated the perceived changes in food habits, appetite and body weight in Tunisian adults of both genders aged 20–74 years old. It should be noted that regarding gender and age, our sample distribution is different from the national distribution characterised by an equal gender distribution for all ages^(22)^. In our sample study, nearly two-thirds of the responses were recorded by women. This predominance has been reported in several other studies^(5–7)^. For age parameter, youth predominance in our sample could be explained by a higher percentage of internet and social network users among adults aged under 35 years old^(23)^. Compared with national statistics^(18)^, the territorial coverage of our sample corresponds to the national distribution for almost all regions, except for the Centre and South-West which were under-represented and Greater Tunis which was by contrast over-represented. This could be justified by a regional disparity of connectivity that probably displayed a concentration of Tunisian Internet users on Greater Tunis with a percentage of 33⋅7 % largely superior to other regions whose percentages are between 7⋅9 and 5⋅4 %^(23)^.

Our survey results showed that more than half of the respondents changed their eating habits, with only 21⋅2 % reporting that the change was positive, i.e. an increase in consumption of fresh fruits, vegetables, pulses, pasta and bread. A comparable percentage (30⋅9 %) of participants reporting improvement in dietary habits was also reported by a large study including participants from the MENA region^(24)^. Negative change i.e. an increase of homemade cakes and biscuits, sweets, processed meat, sugary drinks and alcoholic drinks was reported by a higher percentage of respondents. A recent study reported that food consumption and meal patterns were unhealthier during COVID-19 confinement^(25)^. Along this line, an increase in unhealthy foods and snacks during the COVID-19 home confinement was reported by Bhutani *et al.*^(26)^. Changes in diet and physical activity can lead to an increase in the prevalence of several chronic diseases such as obesity, diabetes that are considered risk factors for mortality in patients with COVID-19^(6)^.

According to our study, appetite gain was predominant in subjects with obesity or at risk of obesity. The addition of meals and excessive snacks during the confinement period was also more prevalent for obese especially those following therapeutic or weight-loss diet population. Laitinen *et al.*^(27)^ reported that stress eating (defined as trying to feel better by eating or drinking in a stressful situation) was significantly associated with obesity, primarily in women. Moreover, stress leads to ‘food craving’ defined as overeating, especially ‘comfort foods’ rich in sugar^(28)^. Those foods, mainly rich in simple carbohydrates, can reduce stress as they encourage serotonin production with a positive effect on mood^(29)^.

Our study showed that men were less likely to change their eating habits towards the negative and had a lower risk of appetite gain and weight gain compared to women. Men also seemed to be less adept at skipping meals, adding snacks and snacking excessively compared to women. There may be a gender-specific response to stress, with women more likely to use food to cope with stress, whereas men are more likely to use other oral behaviours such as drinking or smoking^(30)^. A Spanish longitudinal study carried out during the first periods of the lockdown, showed that women started the confinement with a higher level of negative emotions (stress and avoidance symptoms) than the men group^(31)^. Similarly, Wang *et al.*^(32)^ reported a significant association between gender and high levels of stress, anxiety and depression during the confinement periods.

Our study also showed that retirees were less likely to vary their eating habits and appetite and were the least likely to gain weight. According to Ingram *et al.*^(7)^, an association between changes in occupational status during the pandemic and changes in dietary habits and diet can be established. Indeed, only subjects who did not experience any change in occupational activity during the confinement period were able to maintain their dietary habits stable^(7)^. Furthermore, retirees had the highest EHI score average, while the highest EHD score average was observed in young adults. A possible explanation could be given by the Spanish study which showed that participants aged 18–45 years had higher stress levels during the pandemic period than participants over 60 years^(31)^. The absence of daily routines was more frequent in the youngest group especially students and unemployed people. Their main activity was suddenly interrupted, and many uncertainties appeared around it. Mental status and negative mood may accordingly explain the increased risk of appetite variation either by loss or gain as well as the risk of weight gain associated in our study with the occurrence of a family death from COVID-19 and the fact of living alone^(31,33)^.

It was recently suggested that in times of restrictions due to the COVID-19 pandemic, breaking up prolonged sitting with simple measures, such as alternating between sitting and standing for 30 min periods, may result in meaningful increases in energy expenditure^(34)^. Meanwhile, our study showed that physical activity superior to five times per week presented an inverse association with body weight increase. This result should be utilised for further research and development in public health promotion against sedentary behaviour.

Tunisian adults tend to cook more. The consumption of homemade products such as pastries and bread has increased. By contrast, the consumption of bakery products, sweets, ham, processed meat as well as sweet beverages decreased. Similar changes were observed in the Italian population with an increase in the consumption of homemade desserts, bread and pizza and a decrease in the consumption of soft and sweet drinks along with salty snacks and processed meats^(5)^. Along with healthy food increase consumption, nearby the third of our participants reported a tendency to consume supplementary foods during the pandemic particularly after exposure or infection with SARS-CoV-2. A comparable percentage (26⋅2 %) of multivitamin consumers was reported by Abouzid *et al.*^(24)^ for the MENA population. This was explained by the recommendations of vitamin C^(35)^, vitamin D^(36)^ and Zinc^(37)^ supplementation for COVID-19 prevention and healing.

The present study is the first to be conducted in our country, and thus, it would enrich other studies on the socio-economic impact of the pandemic on Tunisian households. However, the main study limitations are that self-reported data online can be subject to bias and misreporting. In addition, the use of snowball sampling through social media implies that the sample cannot be considered representative of the general Tunisian population, which leads to selection bias. Another limitation is that around one-third of patients who recovered from COVID-19 may have persistent loss of their smell/taste sense^(14)^ and these disturbances may have had impact on the reported dietary changes. Finally, to increase our confidence in the generalisability of the study, it would have to be repeated with the same exercise programme but with different providers in different settings and yield the same results.

## Conclusion

The present study showed that lockdown measures may have increased the stress load and led to an alteration in eating habits and behaviour. Particularly, women, young adults, unemployed subjects along with those living alone were the most vulnerable classes negatively facing this stress. Health promotion campaigns should focus particularly on these subpopulations to limit long-term drawbacks of lockdown measure. To overcome study limitations, it would be interesting in the future to develop stronger tools to investigate eating habits with more confidence.

## Supplementary material

For supplementary material accompanying this paper visit https://doi.org/10.1017/jns.2022.58. click here to view supplementary material
