# Supplementary material for: Impact of COVID-19 lockdown on food habits, appetite and body weight in Tunisian adults
Source: J Nutr Sci. 2022 Jul 13;11:e60. doi: 10.1017/jns.2022.58 (PMC9314320; doi:10.1017/jns.2022.58)
Supplement: Supplementary file 1 [file S2048679022000581sup001.doc]

**Supplementary material 1: Questionnaire**

**Section 1: Sociodemographic data**

| **Questions** | **Answers** |
| --- | --- |
| 1. How old are you? | Age in number |
| 2. Gender | Men/women |
| 3.Nationality | Tunisian/other |
| 4. Place of residence (governorate) | governorate |
| 5. Instruction level | No schooling/ primary schooling/ secondary schooling/ University graduate |
| 6. Occupational status | Unemployed/ housewives/student/worker/ intermediate executive/upper executive/retiree |
| 7. Number of people in the household | Number |

**Section 2: anthropometrics and medical data**

| **Questions** | **Answers** |
| --- | --- |
| 1. Enter your weight (kg) as accurately as possible | Weight in kg |
| 2. Enter you Height (cm) as accurately as possible | Height in cm |
| 3. Do you suffer from any chronic illness? If yes, quote, otherwise write No | Yes/No |
| 4. Do you follow a special diet? (If yes, specify), otherwise write No | Yes/No |
| 5. Did you contract COVID-19? | No/Yes, tested positive with mild form of the disease/ Yes, tested positive with svere form of the disease/ suspected positive / contact case |
| 6. Have you experienced family death due to COVID-19? | Yes/No |
| 7. Have you got a vaccine against COVID-19? | Yes/No |

**Section 3: Dietary habits**

This section focus on your eating habits since the COVID-19 pandemic and more specifically during periods of confinement (first and second quarter of the year 2021).

| **Questions** | **Answers** |
| --- | --- |
| 1. Did your eating habits changed during the  COVID-19 pandemic period? | No/ Yes with increase in consumption of fresh fruits, fish, vegetables, pulses, pasta and bread./Yes with increase in consumption of homemade cakes and biscuits, sweets, processed meat, sugary and alcoholic drinks. |
| 2. During the confinement, which of  these foods are you consuming  MORE than before? | None/fruits/fresh vegetables/frozen vegetables/nuts//fish/frozen  fish/canned fish/legumes/white  meat/pasta and cereals/bread/homemade pizza/homemade pastries/industrial  bakery products/sweets/ham and  processed meat/ red meat /dairy products/  cheese/cow’s milk and yogurt/eggs/coffee, tea, herb tea/sugar or sweeteners/sugary  and sparkling drinks/wine, beer and  alcoholic drinks/snacks/ other |
| 3. During the confinement, which of  these foods are you consuming  LESS than before? | None/fruits/fresh vegetables/frozen vegetables/nuts//fish/frozen  fish/canned fish/legumes/white  meat/pasta and cereals/bread/homemade pizza/homemade pastries/industrial  bakery products/sweets/ham and  processed meat/ red meat /dairy products/  cheese/cow’s milk and yogurt/eggs/coffee, tea, herb tea/sugar or sweeteners/sugary  and sparkling drinks/wine, beer and  alcoholic drinks/snacks/ other |
| 4. Did you change the number of daily meals, during confinement period? | No, it didn’t/Yes, I skip 1 or more of  the main meals (breakfast, lunch,  dinner)/Yes, I skip 1 or more  of snacks between meals/Yes  I added 1 or more of the main  meals/Yes, I added 1 or more of  the snacks between meals/Yes, I  eat out of the meals |
| 5. Did your sense of hunger and satiety change during the period at home for the COVID19 emergency? | No/Yes, less appetite/Yes, more  appetite |
| 6.Did you gain weight during the COVID-19? | No, my weight is stable/ No, I think I  lose weight/Yes, I think I gain not  so much weight/Yes, I think I gain  a lot of weight |
| 7. Since the COVID-19 outbreak, do you consume food supplements? | Yes/No |
| 8. Since the COVID-19 outbreak, do you look for information on nutrition? (if yes, what is your source of information) | No/ Yes in media (TV, Radio..)/Yes in social media / Yes, I'm consulting an expert |
| 9. How many times did you play sports during COVID 19 lockdown? | I didn’t practice sport/1 to 2 times per week/3 to 4 times per week /more than 5 times per week |
